# Modulation of internal solitary waves by the Kuroshio in the northern South China Sea

**DOI:** 10.1038/s41598-023-29931-z

**Published:** 2023-04-13

**Authors:** Yujun Yu, Xu Chen, Anzhou Cao, Jing Meng, Xiaoxin Yang, Tianyang Liu

**Affiliations:** 1 Key Laboratory of Physical Oceanography, Ocean University of China and Qingdao National Laboratory for Marine Science and Technology, Qingdao, 266100 China; 2 Ocean College, Zhejiang University, Zhoushan, 316000 China; 3 PLA Key Laboratory of Hydrographic Surveying and Mapping, Dalian Naval Academy, Dalian, 116018 China

**Keywords:** Physical oceanography, Fluid dynamics

## Abstract

Internal solitary waves (ISWs) in the South China Sea (SCS) are considerably modulated by the background currents. In this study, a three-dimensional high-resolution non-hydrostatic model is configured to investigate how the Kuroshio influences the generation and evolution of ISWs in the northern SCS. Three runs are conducted, including one control experiment without the Kuroshio and two sensitivity experiments with the Kuroshio in different paths. In the Luzon Strait (LS), the Kuroshio reduces the westward baroclinic energy flux radiated into the SCS, resulting in weakened ISWs. In the SCS basin, the background currents further refract the ISWs. With the leaping Kuroshio, the A-waves have longer crest lines but lower amplitudes compared with those in the control run. In contrast, the B-waves are less affected by the leaping Kuroshio. In the presence of looping Kuroshio, the wave refraction caused by the intrusion currents in the SCS basin results in the weakest amplitudes and energy but the widest crest lines of ISWs. Moreover, the energy of the A-waves exhibits double-peak structure along the crest lines. The crest lines of the B-waves extend to 19.5° N, which are more south than those in summer. These results highlight the importance of the Kuroshio on the 3D features of ISWs in the SCS.

## Introduction

Internal solitary waves (ISWs) are ubiquitous in some marginal seas^1^. They often induce strong horizontal velocities and large vertical isopycnal displacements^2–4^, which are threats to underwater structures^5,6^. The breaking of ISWs on topographies causes strong turbulent mixing^7–12^, which further enhances the nutrient flux in the ocean^13^. Hence, understanding the generation, propagation and evolution of ISWs is of vital importance.

ISWs are widespread in the northern SCS. Previous studies indicate that most of ISWs in the northern SCS are originated from the Luzon Strait^2,14–17^ (LS). The strong barotropic tidal currents and steep double ridges at the LS are in favour of the generation of intense internal tides^18^. As the internal tides propagate westward into the northern SCS, they gradually evolve into ISWs due to the combined influence of nonlinearity and dispersion^18,19^. The westward ISWs contain two types: A-wave (rank-order wave packets) and B-wave (single wave). The A-wave occurs at the same time each day, while B-wave occurs about one hour later each day^20^. When the ISWs shoal onto the continental slope, fission and polarity variation may occur^21–24^. Finally, they are dissipated in the shallow water^25^.

Variation of ISWs in the SCS is related to the background processes, e.g., the Kuroshio^3,4,18,26–28^, mesoscale eddies^29–31^ and the SCS western boundary current^32^. The Kuroshio intrusion accompanies variations of background current and stratification. Du et al.^26^ indicated that the enhanced westward tidal currents by the Kuroshio intensify the internal mixing, resulting in stronger ISWs in the SCS. However, Buijsman^18^ and Li^4^ suggested that the ISWs are weakened during the Kuroshio intrusion. Moreover, the variation of the Kuroshio results in the change of strength and inclination of the thermocline in the LS, which affects the generation and evolution of ISWs^27^. The deepening of the thermocline decreases the nonlinearity of internal tides and tends to inhibit the evolution of internal tides into ISWs^28,33^. Li^3^ pointed out that the thermocline tilting increases the growth of westward internal tides, which counteracts the reduction of nonlinearity. Additionally, Li^4^ used a modified two-layer model and clarified the limited effect of thermocline tilting by the Kuroshio on the internal tides. In addition to the Kuroshio in the SCS, mesoscale eddies and the SCS western boundary currents impact the ISWs by modulating wave speed^4,29–32^. These studies deepen our understandings of ISWs in the SCS. However, most of them are based on ideal numerical and theoretical models. To obtain a comprehensive understanding on the modulation of ISWs by the Kuroshio, we need three-dimensional high-resolution numerical simulations, which is the motivation of this study.

The paper is organized as follows. “Methodology” section presents the methodology. The simulation results are shown in “Results” section. A discussion is performed in “Discussion” section. Finally, the paper is summarized in “Summary” section.

## Methodology

**Figure 1:**
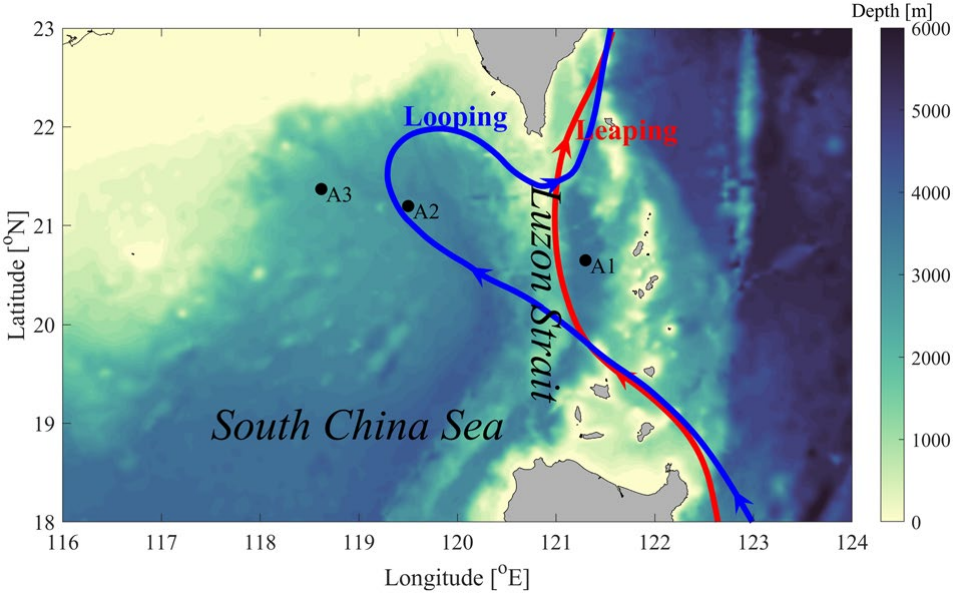
Bathymetry (shading, unit: m) of the northern SCS. The looping and leaping paths of the Kuroshio are denoted by blue and red curves, respectively. Black dots (A1–A3) denote observational stations in Li^3^.

The coastal and regional ocean community model (CROCO, version 1.2, https://www.croco-ocean.org) is used in this study. The model is run in a non-hydrostatic model. The model domain is 116–124° E and 18–23° N, which is shown in Fig. 1. The horizontal resolutions are 1°/240° (approximately 440 m) and 1°/120° (approximately 867 m) along the zonal and meridional directions, respectively, which are comparable to those in previous studies^3,34,35^. In the vertical direction, 45 uneven sigma layers are set, with finer resolutions near the surface and bottom. The horizontal viscosity (diffusivity) is set to 10^–1^ (10^–3^) m^2^/s; while the vertical viscosity (diffusivity) is set to 10^–3^ (10^–5^) m^2^/s. Sponge layers are used at the open boundaries to avoid the reflection of baroclinic waves. The time step is set to 10 s, satisfying the Courant–Friedrichs–Lewy condition.

In the model, bathymetry is extracted from the ETOPO1 dataset^36^. Three runs (Exp0, Exp1 and Exp2) are conducted to explore the effect of the Kuroshio on the generation and evolution of ISWs in the northern SCS. Exp0 is designed as a control experiment, in which the horizontal uniform stratification extracted from the World Ocean Atlas 2009 (WOA09) is used as the initial condition. Considering the variation of the Kuroshio in different seasons (Fig. 1), Exp1 and Exp2 are designed. In Exp1 (Exp2), the time-averaged data from Hybrid Coordinate Ocean Model Reanalysis data (HYCOM, http://tds.hycom.org/thredds/catalog.html) in the summer of 2007 (the winter of 2007) are used as the initial condition and boundary forcing. All the runs are imposed by the same tide forcing (M_2_, S_2_, K_1_ and O_1_) at the open boundaries. Corresponding surface elevations and barotropic currents are predicted by the Oregon University Tidal Inversion Software^37^ (OTIS). All these simulations are operated for 7 days, from 00:00:00 UTC on April 16 to 00:00:00 UTC on April 23, 2007.

## Results

### Model validation

**Figure 2:**
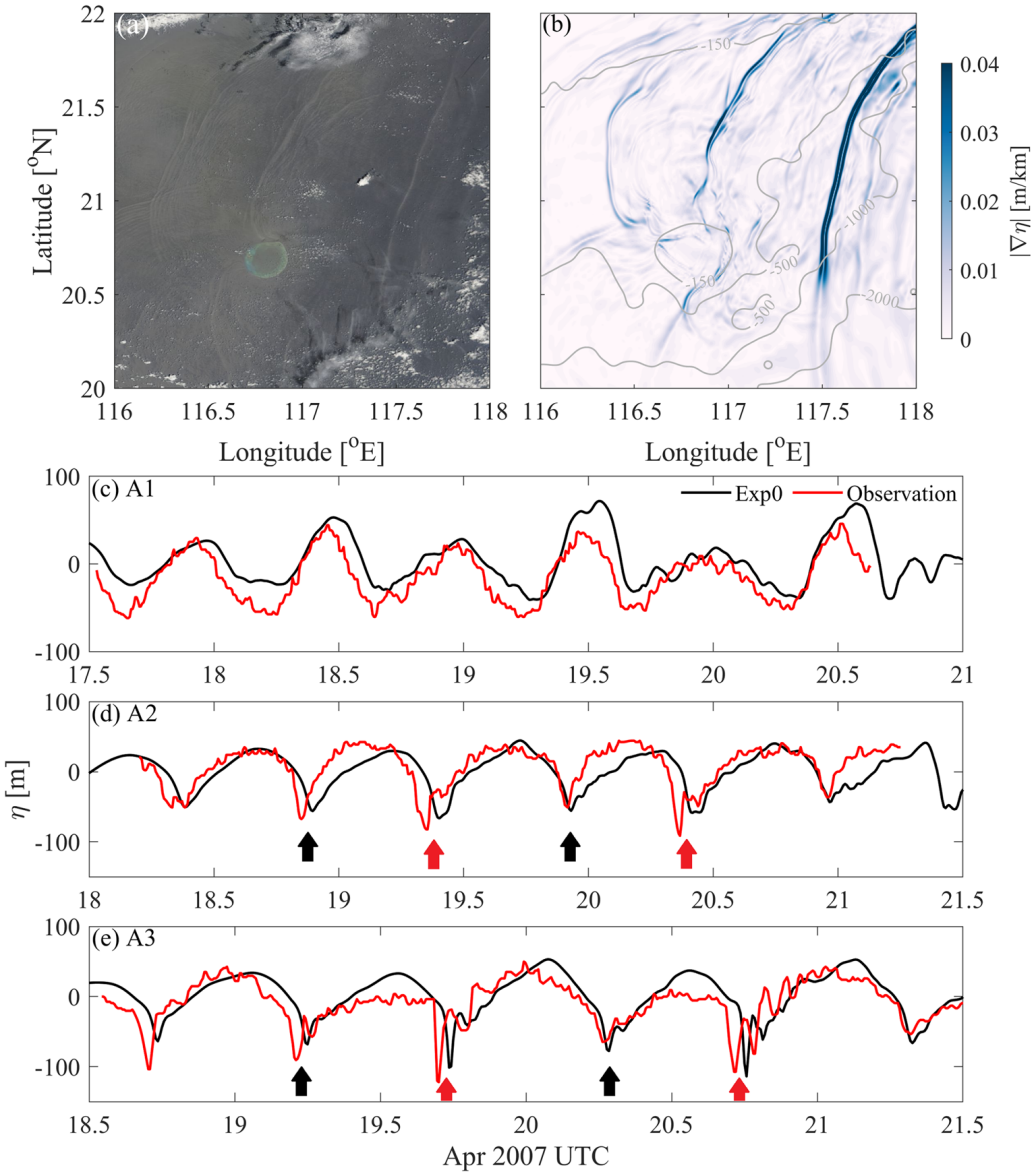
(**a**) MODIS image at 03:00:00 April 20, 2007, UTC. (**b**) The gradient of the sea surface height (shading, unit: m/km) in Exp0 at 03:00:00 April 20, 2007, UTC. Grey contours in the subfigures denote the isobaths Comparisons between observations3 and model results of Exp0 at (**c**) A1, (**d**) A2 and (**e**) A3. In (**d,e**), the red and black arrows indicate the A-waves and B-waves, respectively.

The simulated results are validated by comparing with satellite observations. The surface convergence and divergence zones included by ISWs lead to variations of sea surface roughness. Following Gong^38^, we calculated the gradient of sea surface height $|\nabla \eta |$, from which the wavefronts are detected. In Fig. 2a, three ISWs radiate westward from the LS, which are located at 117.5-118° E, 116.8-117.5° E and 116.3-116.5° E, respectively. The simulated ISW fronts basically appear at the same locations as those found in the MODIS image, and they have a similar vertical structure to the observational ones (Fig. 2a,b). This result implies the accuracy of our simulated results.

Then the simulated isotherm displacements are compared with in-situ observations from Li^3^. As shown in Fig. 2b, periodic signals are found at the A1 in the LS, which are strong internal tides. At A2 and A3 in the SCS basin, both the simulated and observational results indicate alternative occurrences of A-wave and B-wave. The amplitudes of simulated ISWs are generally consistent with those observed at A2 and A3. The averaged speed of the A-waves (B-waves) is 4.1 m/s (4.0 m/s) from A2 to A3 in both Exp0 and observations. However, there are slight differences in the phases of ISWs between simulated results and observations, which may be attributed to the lack of background currents and horizontally varying stratification.

**Figure 3:**
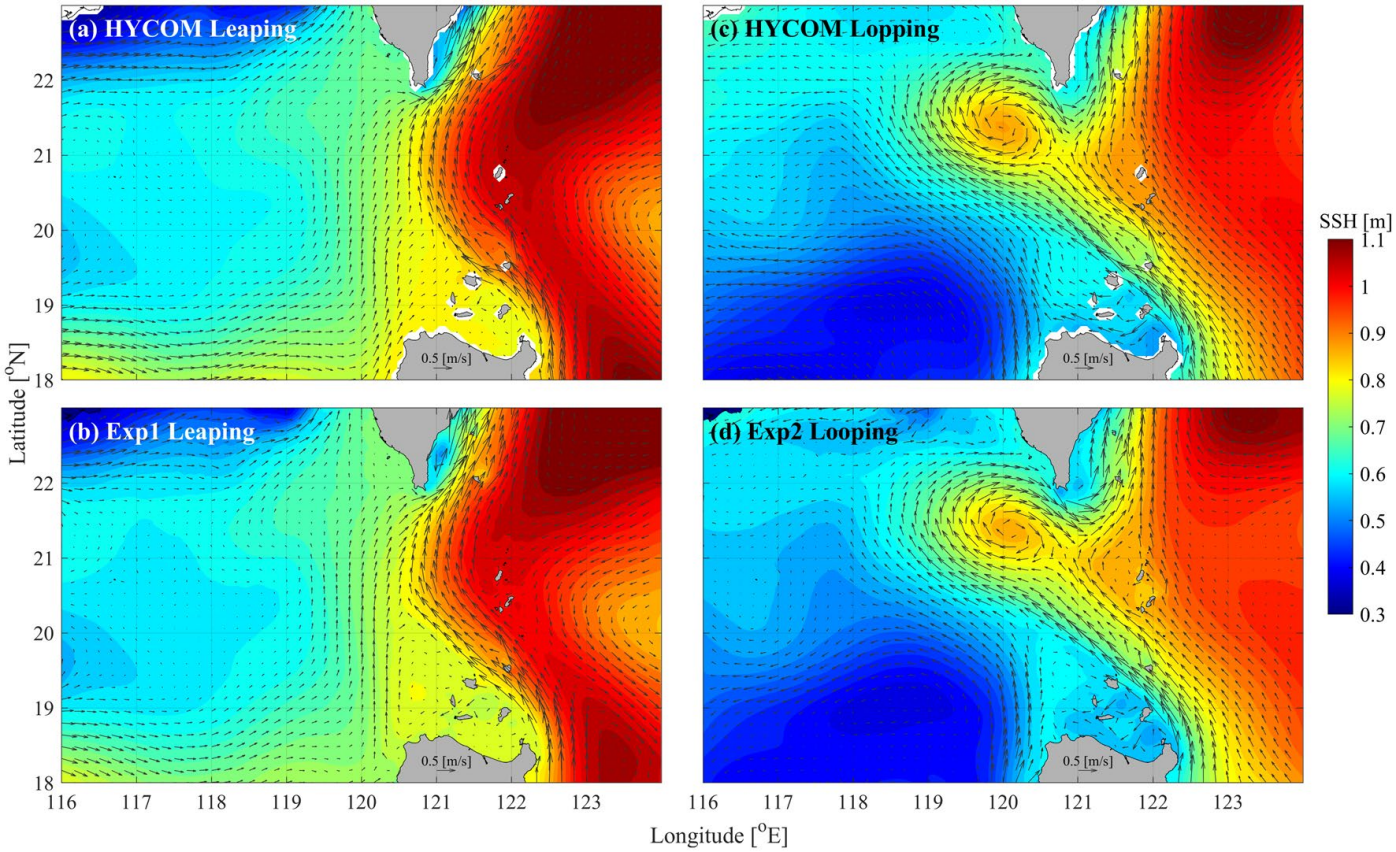
Comparisons of averaged sea surface height (shadings, units: m/s) and velocity (black arrows, unit: m/s) between (**a,c**) HYCOM data and (**b,d**) model results.

The simulated background currents are validated by comparing with those from the HYCOM Reanalysis data. As shown in Fig. 3, the simulated Kuroshio agrees well with those from the HYCOM results. In Exp1, the Kuroshio takes a leaping path^39^ and no pronounced intrusion into the northern SCS occurs. While in Exp2, the Kuroshio takes a looping path and intrudes into the northern SCS in the middle of LS, forming an anti-cyclonic loop (Fig. 3c,d). Based on the above results, we believe that the simulated results are reasonable, which can be used to investigate the influence of the Kuroshio on the ISW.

### Wave characteristics

**Figure 4:**
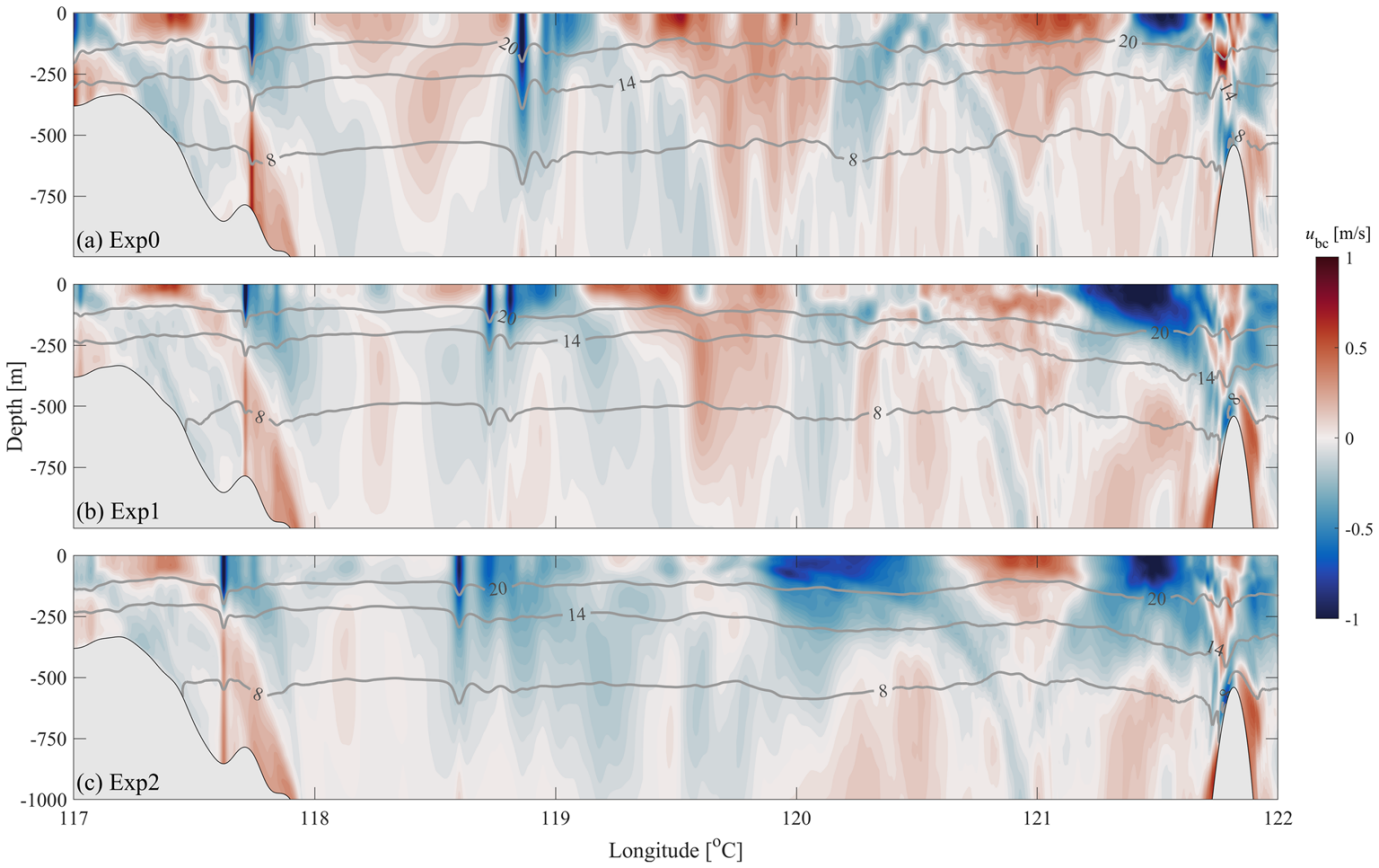
The zonal baroclinic velocities (shadings, units: m/s) of (**a**) Exp0, (**b**) Exp1 and (**c**) Exp2 along the section in Fig. 2 at 14:00:00 April 19, 2007, UTC. Grey contours represent isotherms.

Figure 4 shows the vertical transect (Black lines in Fig. 2a) of zonal baroclinic velocities. The beams of internal tides generated from the east ridge of LS are found, and no obvious ISW occurs near the east ridge in Fig. 4. There are A-wave and B-wave located at 118.9° E and 117.7° E in Exp0 (Fig. 4a), respectively. Under the influence of the Kuroshio, the ISWs in Exp1 and Exp2 have smaller amplitudes but move faster than those in Exp0 (Fig. 4).

**Figure 5:**
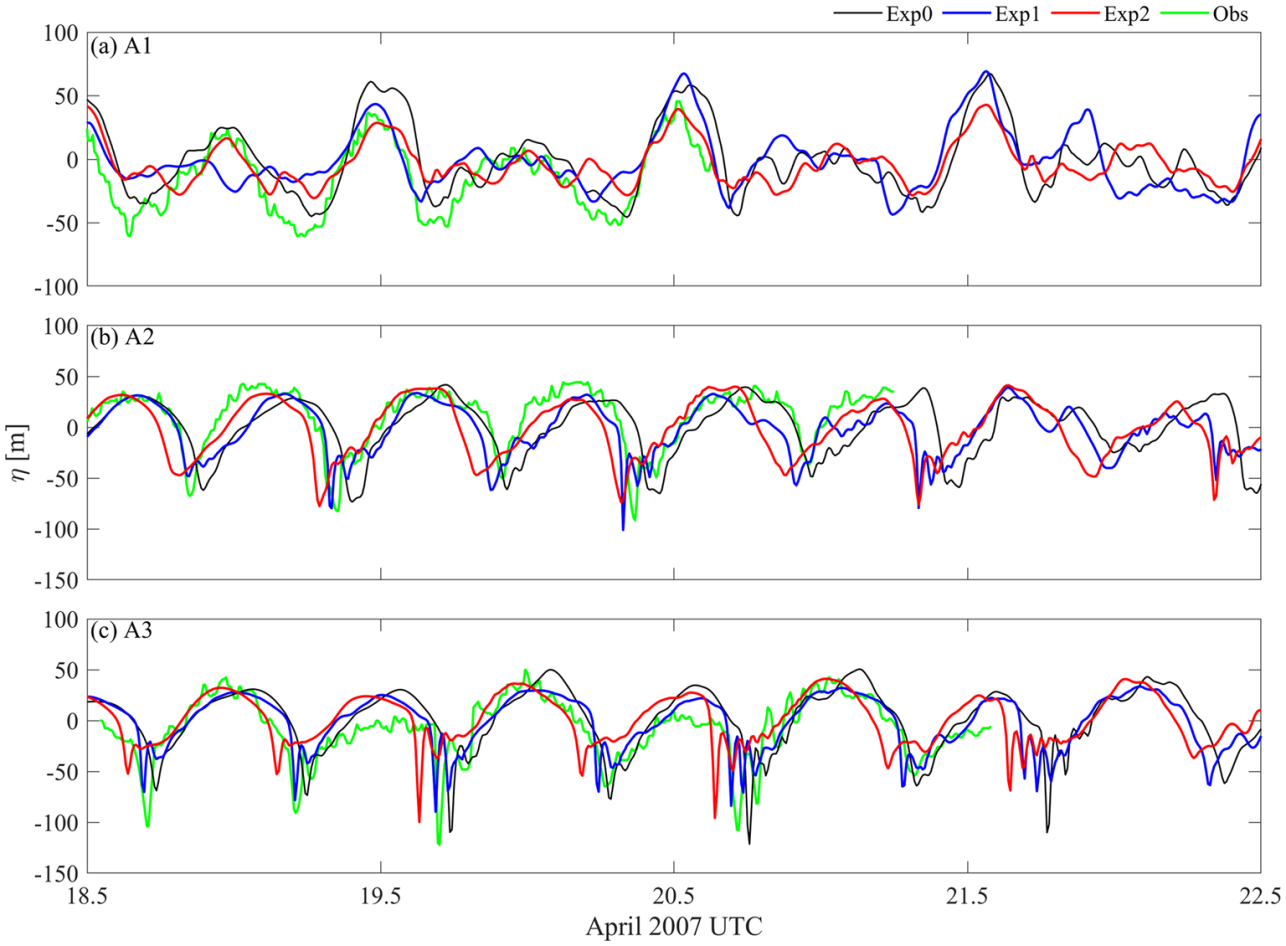
Time series of 9 °C isotherm displacement (units: m) of Exp0 (black lines), Exp1 (blue lines), Exp2 (red lines) and observations in Li (2014, green lines) at (**a**) A1, (**b**) A2 and (**c**) A3 (shown in Fig. 3a).

The time series of isotherm displacements at A1–A3 are shown in Fig. 5. At A1 in the LS, the internal tide has the same phase in the three runs, while the amplitude of the internal tide in Exp2 is a little smaller (Fig. 5a). This may be due to the smaller tidal conversion under the looping Kuroshio^40^. In the presence of the Kuroshio, the A-waves in Exp1 and Exp2 evolve into the rank-ordered wave packets at A2, with averaged amplitudes of 77 m and 75 m, respectively. In contrast, the A-waves have the waveform of a single wave at A2 in the absence of the Kuroshio, with an averaged amplitude of 63 m. The averaged amplitudes of the B-waves in three runs are 57, 56 and 47 m, respectively. In Exp2, the B-waves have the waveform of a corner wave^41^, with the smallest amplitudes. At A3 (Fig. 5c), the averaged amplitudes of the A-waves (116 m) and B-waves (72 m) in Exp0 are larger than those in Exp1 (72 m and 71 m) and Exp2 (84 and 51 m). Moreover, the Kuroshio increases the moving speed of ISWs, especially for the looping Kuroshio in Exp2.

**Figure 6:**
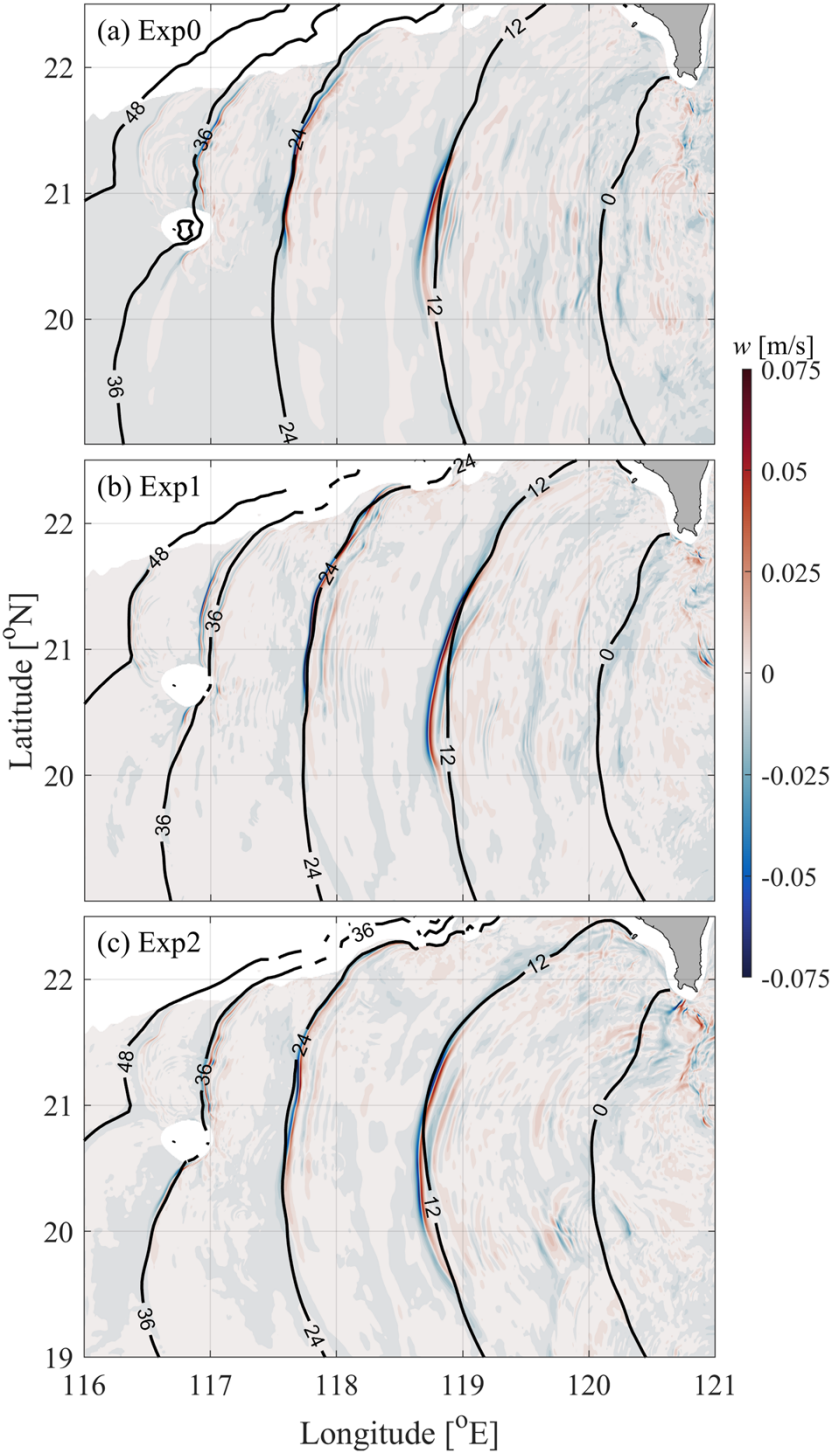
Snapshots of vertical velocity (shadings, units: m/s) at z = -150 m for (**a**) Exp0, (**b**) Exp1 and (**c**) Exp2. The black lines show the wavefronts derived from the wavefront tracing model considering *U* and *N*, noting that the *U* = 0 in Exp0.

Wavefronts of ISWs are shown by vertical velocities at *z* =  − 150 m (Fig. 6). During the westward propagation of ISWs, there are convex crest lines in the northern SCS. Compared with Exp0, the crest line of ISWs near 118.5° E in Exp2 is longer and significantly refracted, which is due to the looping Kuroshio. In contrast, the crest lines of ISWs near 118.5° E in Exp0 and Exp1 are slightly different, because of the weak intrusion of the Kuroshio in Exp1. As a result, the arrival time of ISWs at A2 and A3 is different in the three runs, especially in Exp2 (Fig. 5).

The empirical wavefront tracing model from Jackson^42^ is employed in this study. In this model, the travel time for a propagating wavefront is obtained by solving the eikonal differential equation,

1 $$\left( {\frac{\partial T}{{\partial x}}} \right)^{2} + \left( {\frac{\partial T}{{\partial y}}} \right)^{2} = \frac{1}{{c^{2} }},$$

where *T*(*x*, *y*) represents the travel time between the origin point and a given location. The wave speed *c*(*x*, *y*) is calculated by the T-G equation,

2 $$\hat{w}_{zz} + \left[ {\frac{{N^{2} }}{{(U - c)^{2} }} - |k|^{2} - \frac{{U_{zz} }}{U - c}} \right]\hat{w} = 0,$$

where $\hat{w}$ is the eigenfunction of vertical velocity, *U* is the background current, *N* is the buoyancy frequency, and *k* is the horizontal wave number. For each run, the wavefront tracing model is employed with wave speed *c* considering both *U* and *N* (*U* = 0 in Exp0). When the wave speed is obtained, the wavefronts can be predicted according to Eq. (1). As shown in Fig. 6, the wavefront tracing model results basically agree well with the simulated results.

### Wave intensity

**Figure 7:**
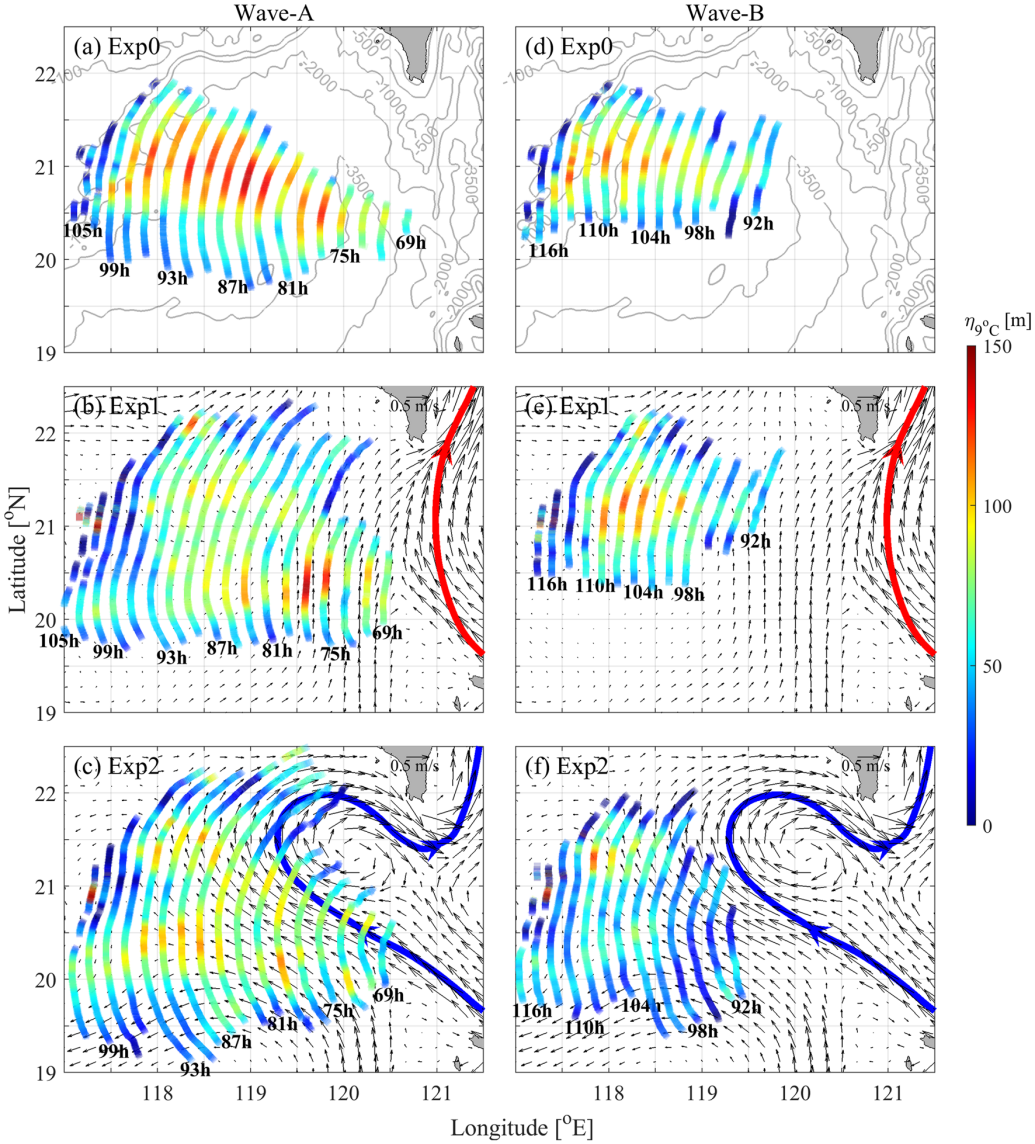
Wave amplitudes (shadings, units: m) along the crest lines for the A-wave and B-wave in Exp0 (**a,d**), Exp1 (**b,e**) and Exp2 (**c,f**). Contours in (**a,d**) denote the bathymetry. Surface background currents are indicated by black arrows in (**b,c,e,f**). The leaping and looping paths of the Kuroshio are marked by red and blue curves, respectively.

The amplitudes of the A-wave and B-wave in each run are shown in Fig. 7. In Exp0, with the westward propagation (approximately 17° west by north from the LS), the A-wave amplitudes vary from 90 to 140 m, and the B-waves amplitudes vary from 60 to 120 m, a little smaller than those of the A-wave (Fig. 7a,d).

In Exp1 (Fig. 7b,e), due to the effect of the Kuroshio on the baroclinic tides from the LS, the original crest line of A-wave in Exp1 at 69 h is different from that in Exp0, resulting in further differences in the evolution of ISWs. In the SCS basin, the crest lines of the A-wave extend north to 22.3°N and south to 19.7° N. In addition, the maximum amplitude in each crest line moves south to approximately 20.4° N, ranging from 80 to 140 m. The amplitudes of the B-wave are slightly smaller (50–120 m), but the crest lines are slightly longer, compared with Exp0.

In Exp2 (Fig. 7c,d), the crest lines of ISWs are the longest and have the smallest amplitudes. The crest lines of the A-wave range from 19.2 to 22.5° N, with double peaks of amplitude (60–110 m), of which one is nearly along the looping path of the Kuroshio. The crest lines of the B-wave extend to 19.5° N, which is approximately 1° more south than those in Exp0 and Exp1. At the same time, the B-wave has smaller amplitudes (50–110 m).

**Figure 8:**
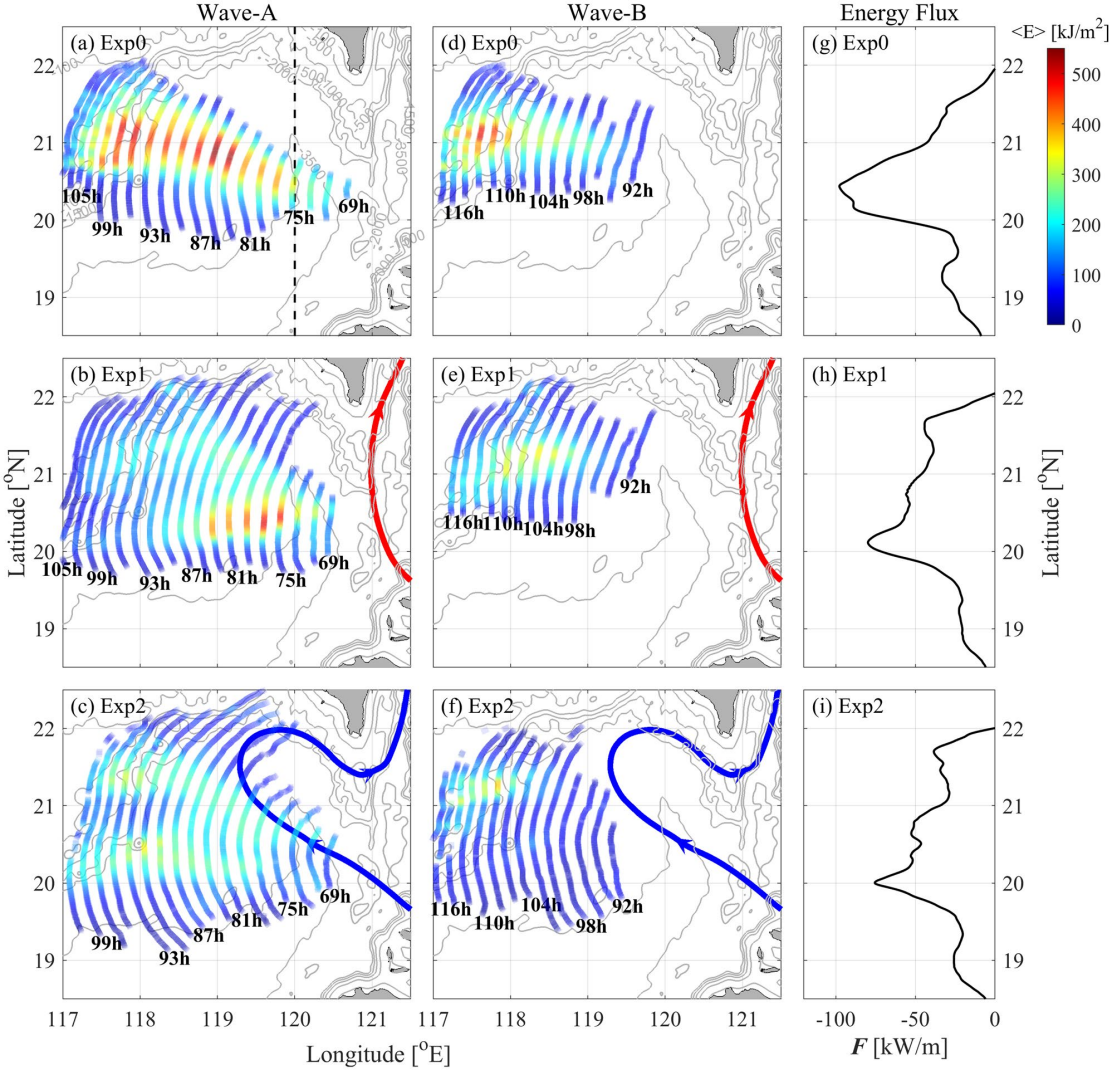
Wave energy (shadings, units: kJ/m^2^) along the crest lines of the A-wave and B-wave in (**a,d**) Exp0, (**b,e**) Exp1and (**c,f**) Exp2. Depth-integrated zonal baroclinic energy flux along 120°E (black dashed line in **a**) are shown in (**g–i**). Grey contours in the subfigures denote the isobaths. The leaping and looping paths of the Kuroshio are marked by red and blue curves, respectively.

The energy of the A-wave and B-wave in each experiment is shown in Fig. 8a–f. The vertically integrated energy is given by

3 $$E{ = }\int_{ - H}^{0} {(KE + APE)dz} ,$$

where

4 $$KE{ = }\frac{{\rho_{0} }}{2}\left( {u^{^{\prime}2} + v^{^{\prime}2} + w^{2} } \right),$$

is the baroclinic kinetic energy density, in which *u*' and *v*' are the horizontal baroclinic velocity components, *w* is the vertical velocity, and *ρ*_0_ is the reference density. The APE is calculated as^23,43^

5 $$APE = \frac{{g^{2} \rho^{^{\prime}2} }}{{2\rho_{0} N^{2} }} + \frac{{g^{3} (N^{2} )_{z} \rho^{^{\prime}3} }}{{6\rho_{0}^{2} N^{6} }} + O\left( {\rho^{^{\prime}4} } \right),$$

where *ρ*′ is the density perturbation induced by the wave motion. The integral westward energy flux at 120° E (dotted line in Fig. 8a) in each experiment is shown in Fig. 8g–i, which is represented by

6 $$\vec{F} = p \cdot \overrightarrow {u^{\prime}} ,$$

where *p* is the pressure perturbation satisfying the baroclinic conditions^44^,

7 $$\frac{1}{H}\int_{ - H}^{0} {pdz = 0} .$$

In Exp0, the westward energy flux from the LS has a peak of 97.6 kW/m (Fig. 8g), and the energy of ISWs is mainly concentrated on section roughly 17° west by north from the LS, decaying rapidly to north and south along the crest lines (Fig. 8a,d). The energy of the A-wave is larger than that of the B-wave. For the sensitivity runs Exp1 and Exp2, the westward energy fluxes are redistributed by the Kuroshio. The maximal values are 79.8 kW/m and 75.5 kW/m, which are decreased by 18% and 23% compared with that in Exp0, respectively (Fig. 8h,i). In Exp1, for the A-wave, the energy is lower and divergent in comparison to Exp0 (Fig. 8b). The B-wave basically shares a similar pattern to that in Exp0, with a bit smaller energy but longer crest lines. In Exp2, the wave energy is further weakened (Fig. 8c,f), because the westward energy flux into the SCS is the weakest among the three runs (Fig. 8i). However, both the A-wave and B-wave have much longer crest lines than those in Exp0 and Exp1.

**Figure 9:**
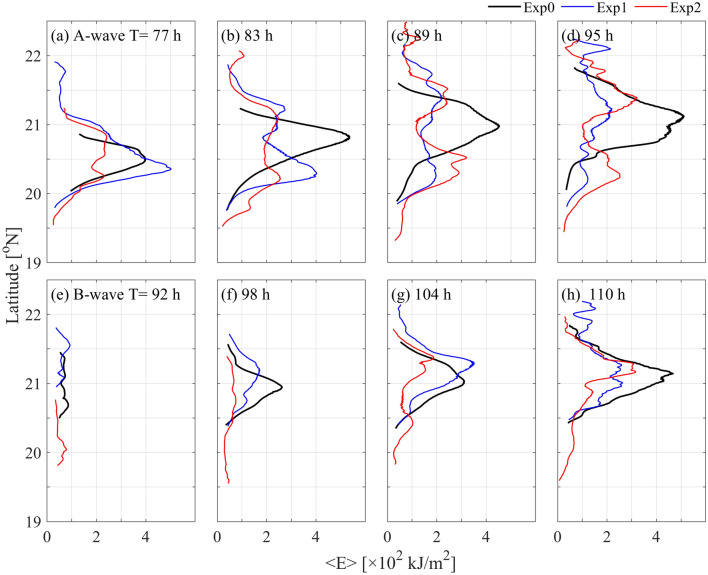
Meridional distribution of energy for (**a–d**) the A-wave and (**e–h**) B-wave at different times.

Figure 9 shows the meridional distribution of wave energy. In Exp0, there is a significant peak in the energy of the A-wave, which gradually shifts northward as the A-wave propagates westward across the SCS basin (Fig. 9a–d). Similar feature is also found for the B-wave, although its energy is lower than that of the A-wave (Fig. 9e–h). Under the influence of the Kuroshio, the crest lines of both the A-waves and B-waves are stretched. In Exp1, the maximal value of the A-wave energy is located at 20.4° N in each running time of 69–89 h (Fig. 9a–c, 118.3–120.3° E in Fig. 8b). Double peaks of wave energy appear as the A-wave further propagates into the SCS basin. In contrast, the meridional distribution of the B-wave energy seems to be less influenced by the Kuroshio, since the multi-peak pattern is only found as the wave approaches the continental slope (118.0° E). With the looping Kuroshio, the A-wave energy shows double peaks of energy even near the LS (Fig. 9a,b). Similarly, the B-wave energy also has double peaks, although one peak is lower than the other.

## Discussion

In the LS, the westward energy flux is decreased in the presence of the Kuroshio (Fig. 8g–i). In Exp1 and Exp2, the meridionally integrated westward energy fluxes at 120.5° E are 15.2 GW and 14.0 GW, respectively, which are 4% and 12% smaller than that in Exp0 (15.9 GW). The Kuroshio in the LS suppressed the generation of the internal tides^4^, resulting in the reduction of westward baroclinic energy flux (Fig. 8g–i), which further influences the generation of westward ISWs from the LS (Fig. 9a,e). As the ISWs go into the SCS basin, their propagation and intensity are further modulated by the Kuroshio currents, especially under the looping Kuroshio (Figs. 7, 8).

**Figure 10:**
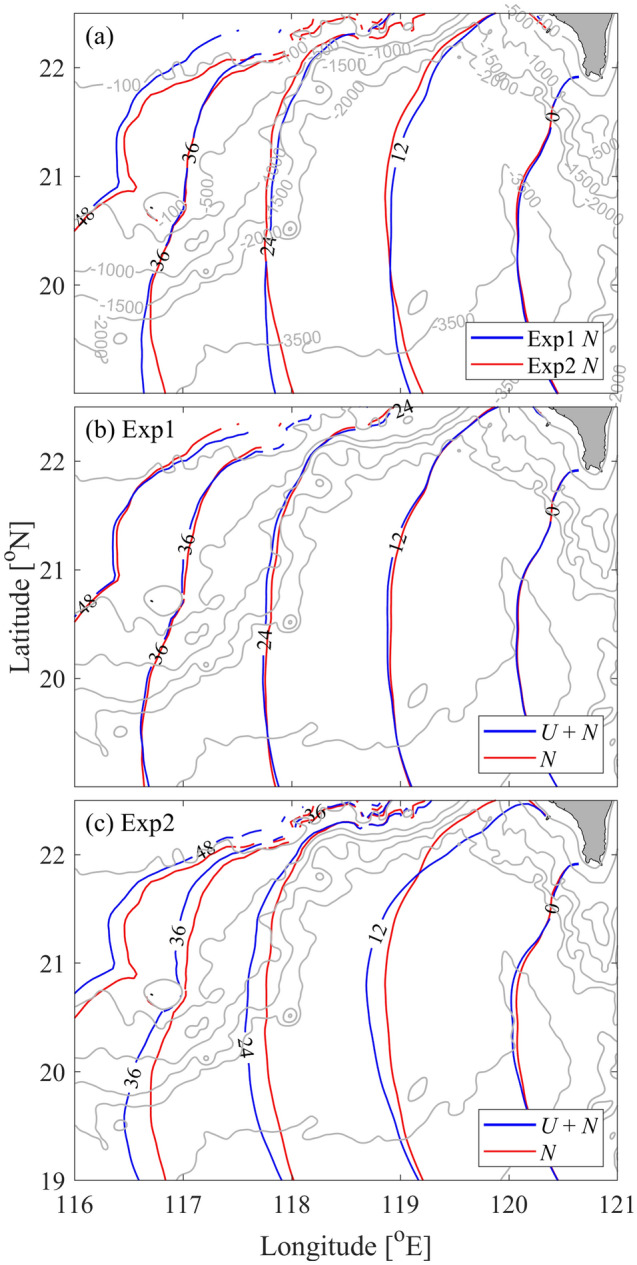
(**a**) The predicted wavefronts without considering the background current. (**b,c**) Comparison of wavefronts calculated by considering only *N* and both *U* and *N* for Exp1 and Exp2.

According to Eq. (2), both the stratification and current can modulate the wave speed and hence affect the propagation of ISWs. In this study, the wavefront tracing model is used to explore the effect of stratification and current on the ISWs in the basin of SCS. Additional calculations on wave speed by only considering *N* are carried out. As shown in Fig. 10a, the variation in seasonal stratification has a minor influence on the ISWs. Neglecting the background current *U*, the predicted wavefronts in Exp2 show large differences from those considering both *U* and *N* (Fig. 10c). This result highlights the influence of background currents on modulating the propagation of ISWs. However, in Exp1, the wavefronts predicted with both *U* and *N* show small differences from those predicted only with *N* (Fig. 10b), which is largely due to the weak background currents in the SCS basin (Fig. 3).

In this study, how the Kuroshio influences the westward propagating ISWs from the LS is investigated. However, in the SCS basin, there exist other processes (e.g., mesoscale eddies and boundary currents) that modulate the ISWs^4,31,32^. Although progress has been made by several previous studies based on theory and observations^4,28,30–32^, 3D high-resolution simulations with realistic forcing would provide comprehensive insights. Moreover, note that modulation of background currents on ISWs also occurs in other regions of the ocean, such as, the Amazon shelf^33^, the Portuguese shelf^45^, the Western Iberian Shelf^46^ and the Andaman Sea^47^. Results of this study may be helpful for understanding similar processes in these regions.

## Summary

In this study, we use a three-dimensional non-hydrostatic model to investigate the effect of the Kuroshio on the generation and evolution of ISWs in the northern SCS. Two sensitivity runs considering the Kuroshio in summer and winter are conducted. In addition, the wavefront tracing model is employed to explore the effect of background currents on the evolution of ISWs. The conclusions are listed as follows:

- The Kuroshio plays an important role in the modulation of ISWs in the northern SCS. In the LS, internal tide generation is suppressed in the presence of the Kuroshio, causing less energy radiated into the SCS and hence the weakened ISWs. The background currents of the Kuroshio in the SCS basin refract the ISWs, resulting in the redistribution of the energy along the crest line of ISWs.
- In the presence of leaping Kuroshio, the suppressed ISWs are radiated from the LS into the SCS basin. The A-waves spread out with longer crest lines but weaker energy and amplitudes. In contrast, the B-waves are less affected by the leaping Kuroshio.
- When the Kuroshio takes a looping path, the strongest westward inflow reduces the westward baroclinic energy flux by 12% compared with that without Kuroshio. The background currents of the looping path in the SCS basin further refract the westward ISWs in the SCS basin. As a result, the ISWs are weaker and have longer crest lines than that in summer, especially for the B-waves. Moreover, the energy of the A-waves has double peaks along the crest lines.

## Data Availability

Bathymetry is obtained from https://ngdc.noaa.gov/mgg/global/. The WOA09 data are downloaded from https://climatedataguide.ucar.edu/climate-data/world-ocean-atlas-woa09. The HYCOM reanalysis data are downloaded from http://tds.hycom.org/thredds/catalog.html. The OTIS is downloaded from https://www.tpxo.net/. The source code of CROCO (version 1.2) is downloaded from https://www.croco-ocean.org. The MODIS image is downloaded from https://go.nasa.gov/3WvlbuH.
